# The Oriental flat bug genus *Libiocoris* Kormilev, 1957 revisited: re-examination, synonymy, and description of a new genus (Heteroptera, Aradidae)

**DOI:** 10.3897/zookeys.789.26165

**Published:** 2018-10-10

**Authors:** Xiaoshuan Bai, Ernst Heiss, Wanzhi Cai

**Affiliations:** 1 Institute of Life Science and Technology, Inner Mongolia Normal University Huhhot China; 2 Inner Mongolia Normal University, Zhaowuda Road 81, Tiroler Landesmuseum Innsbruck Austria; 3 Huhhot, 010022, Inner Mongolia, China China Agricultural University Beijing China; 4 Entomological Research Associate, Inner Mongolia Normal University Huhhot China; 5 Tiroler Landesmuseum, 2a Josef Schraffl Strasse, A-6020 Innsbruck, Austria Tiroler Landesmuseum Innsbruck Austria; 6 Department of Entomology, China Agricultural University, Yuanmingyuan West Road, Beijing, 100094, China China Agricultural University Beijing China

**Keywords:** Aradidae, Carventinae, China, Heteroptera, *
Libiocoris
*, new combination, new genus, new species, new synonymy, *
Paralibiocoris
*

## Abstract

Re-examination of type specimens of *Libiocoris*Kormilev 1957, *L.poecilus* from New Guinea and other taxa assigned to this genus, the Chinese *Libiocorisheissi* Bai, Yang & Cai, 2006 and *Libiocorissinensis* Bai, Yang & Cai, 2006 proved to be synonyms, thus *L.heissi* = *L.sinensis* syn. n. They are, however, different from *Libiocoris* Kormilev, 1957 to which they were originally assigned and a new genus *Paralibiocoris***gen. n.** is proposed for them. Therefore *Paralibiocorisheissi* comb. n. = *Libiocorisheissi* Bai et al., 2006 = *Libiocorissinensis* Bai et al., 2006, syn. n. From Hainan Island, China, the following new species, *P.roundangulus***sp. n.**, *P.hainanensis***sp. n.**, and *P.limuensis***sp. n.**, are described and figured and a key to species is provided.

## Introduction

The genus *Libiocoris* was erected by Kormilev, 1957 for the species *poecilus* from New Guinea. Usinger & Matsuda, 1959 improved the generic description based on the new species *L.antennatus* from Papua (New Guinea) and added *L.angulatus*, also from New Guinea to this genus. Later, *L.lobatus* Kormilev, 1968 and *L.pilicornis* Kormilev, 1972 were described again from New Guinea. Heiss (1982) described the species *L.indicus* from north India and two more species, *L.heissi* and *L.sinensis*, were described by Bai et al. (2006) from Hainan Island in south sast China.

The distribution pattern of these eight species within the Indo-Pacific region seems restricted to two biogeographically different areas: the Indo-China region (*L.indicus*, *L.heissi*, and *L.sinensis*) and the Papuasian region (*L.poecilus*, *L.antennatus, L.angulatus*, *L.lobatus*, and *L.pilicornis*), which raises questions about their assignment to the same genus of apterous Carventinae, all having very limited distribution ranges.

As a result of the re-examination of the genus-type species *L.poecilus* (holotype male, allotype female, HMHN), of *L.angulatus* (holotype female, MCSM), and of *L.antennatus* (paratype female, CEHI ex. coll. Kormilev), the original descriptions of *L.lobatus* and *L.pilosus* as well as the types of other species assigned to this genus, we can now confirm the following results:

1 After removal of the waxy incrustation obscuring the dorsal structures and examination of the female holotypes of *heissi* and *sinensis* it was evident that both belong to the same taxon and are synonyms.

2 The Chinese species *heissi* and *sinensis* differ in essential morphological characters from *Libiocoris* sensu Kormilev, 1957, for which a new genus *Paralibiocoris* gen. n. is proposed. A further three new species from China belonging to this genus are recognized and described herein.

3 The single species *L.indicus* Heiss, 1982 described from north India, tentatively assigned to *Libiocoris*, is not congeneric with *Libiocoris* sensu Kormilev, 1957 nor to *Paralibiocoris* gen. n. erected for the Chinese species.

4 Inconsistencies and remarkable differences in the descriptions of *angulatus*, *antennatus*, *lobatus*, and *pilosus* raise questions about their congeneric assignment when compared with *poecilus*.

## Materials and methods

Depositories of type material examined:

**MNHUK** Museum of Natural History, London, Great Britain

**CAU** China Agricultural University, Beijing, China

**CEHI** Collection Ernst Heiss, Tiroler Landesmuseum Innsbruck, Austria

**EMIH** Entomological Museum of Inner Mongolia Normal University, Huhhot, China

**HNHM** Hungarian Natural History Museum, Budapest Hungary

**MCSM** Museo Civico di Storia Naturale “Giacomo Doria”, Genoa, Italy

**MHNG** Muséum d’ Histoire Naturelle, Geneva, Switzerland

Photographs were taken through Keyence VHX-1000 equipment. Measurements were made using a calibrated micrometre; all measurements are given in millimetres. Abbreviations used as follows:

**deltg** dorsal external laterotergite (connexivum);

**mtg** mediotergite;

**ptg** paratergite;

**vltg** ventral laterotergite.

## Taxonomy

### Synonymy

Type specimens of *L.heissi* and *L.sinensis* are conspecific, thus the following synonymy *Libiocorisheissi* Bai, Yang & Cai, 2006: 41 = *Libiocorissinensis* Bai, Yang & Cai, 2006: 43 syn. n. is here established, *heissi* having priority.

#### Paralibiocoris gen. n.

Taxon classificationAnimaliaHemipteraAradidae

http://zoobank.org/BE3C5C3E-DE96-418B-A434-A60EBC5E0FB8

##### Type species.

*Libiocorisheissi* Bai, Yang & Cai, 2006.

##### Diagnosis.

**Figures 1–4.**
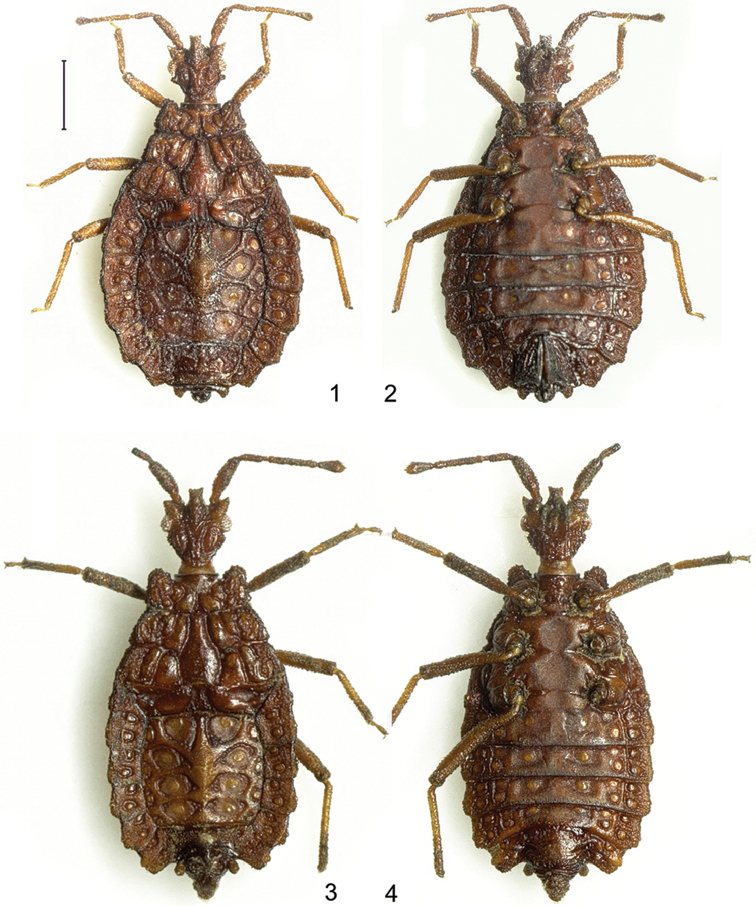
*Paralibiocorisheissi*. Holotype female (**1, 2**) dorsal and ventral view; male (**3, 4**) dorsal and ventral view. Scale bar: 1 mm.

**Figures 5–17.**
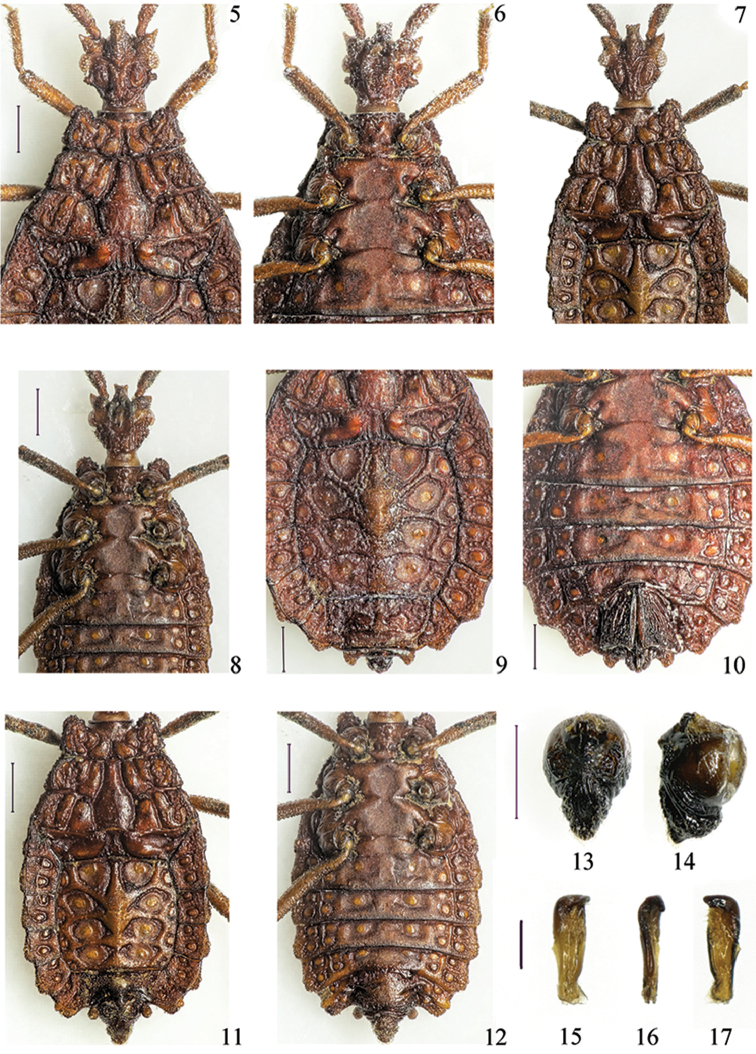
*Paralibiocorisheissi*. Holotype female (**5, 6, 9, 10**) dorsal and ventral thorax and abdomen; male (**7, 8, 11, 12**) dorsal and ventral thorax and abdomen; pygophore dorsal and lateral view (**13, 14**); right paramere in three positions (**15, 16, 17**). Scale bars: 0.5 mm (**5–14**), 0.1 mm (**15–17**).

General aspect similar to *Libiocoris* Kormilev, 1957 but is distinguished from the type species *Libiocorispoecilus* (characters in brackets) by the following set of morphological characters:

• position of spiracles: II ventral, III–VII lateral and visible from above (II–III ventral IV–V sublateral not visible from above, VI–VII lateral and visible);

• fused deltg II+III shorter, reaching only posterior border of metanotum (Figs 1, 3) (extending forward to half-length of mesonotum which is not shown in Fig. 1 of Kormilev’s (1957) description but mentioned by Usinger and Matsuda’s (1959) redescription, and verified at types (Figs 81, 82);

• presence of a smooth oblique callus on vltg VII of male which is independent of spiracle VII (Figure 12) (lacking and not developed, fig. 4 of Kormilev 1957);

• fused median longitudinal sclerite reaching from pronotum to tergal plate bottle-shaped along meso- and metanotum, then restricted along mtg I+II and carinate, the fusion line between metanotum – mtg I+II marked by a suture (Figs 5, 7) (narrow and subparallel along meso-metanotum with a longitudinal sulcus, fused to but without a suture between metanotum – mtg I+II) (Figs 81, 82);

• median ridge of abdomen distinctly elevated along midline (flat, not developed), dorsally reflexed vltg VII subrectangular (produced posteriorly, long and acute in male, shorter and acute in female);

• shape of male pygophore pyriform, produced posteriorly (wide and short).

*Paralibiocoris* gen. n. is very similar to *Bruneiaptera* Heiss, 2011 from Borneo, sharing basic habitus and dorsal thoracic structures; however, in *Brunneiaptera* all spiracles (II–VII) are lateral and visible from above.

##### Description.

Apterous, of small size 4.4–5.8 mm; habitus elongate-oval; legs and antennae beset with small setigerous granules; coloration yellowish to reddish or blackish brown.

*Head.* Subquadrangular, longer or as wide as distance across eyes; clypeus short, genae slightly produced; antenniferous tubercles short with acute apices; antennae long and slender, first and third and second and fourth segments subequal in length, first stout, incrassate, second and third cylindrical, fourth fusiform; eyes small, granulate; postocular tubercles distinct; rostrum arising from a slit-like atrium, not reaching limits of rostral groove.

*Thorax.* Pronotum short and wide; anterolateral angles produced forward beyond collar forming large blunt or rounded lobes; disc with a median sulcus; separated from mesonotum by a transverse intersegmental furrow; meso- and metanotum separated only laterally, the elevated median ridge smooth without sulcus; lateral sclerites with longitudinal elevations; metanotum separated from fused mtg I+II by a narrow transverse sulcus;

*Abdomen.* Mtg I and II fused together; mtg III to VI fused into a subquadrangular tergal plate, elevated along midline with usual pattern of large and small callous spots and dots; mtg VII strongly elevated posteriorly in male and slightly elevated in female; pygophore cordate; paratergites VIII clavate or lobiform.

*Venter.* Prosternum raised and with Y-shaped median carina; meso- and metasternum and sternum II+III fused and flattened medially. Spiracles II ventral, III-VII lateral on dorsally reflexed vltg III-VII and visible from above; spiracle VIII terminal on ptg VIII.

*Legs.* long and slender, without spine, preapical comb on fore tibia present, femora subcylindrical, claws with fine pulvilli.

##### Etymology.

From “para-“ close to (Greek) and *Libiocoris*.

### Key to species *Paralibiocoris* gen. n. from China.

**Table:** 

1	Antennal segment I as long as III	**2**
–	Antennal segment I longer than III	**3**
2	Antennae longer, 2.1 times as long as width of head, anterolateral lobes of pronotum narrow and produced (Figs 1, 3), abdomen of female egg shaped, widely rounded (Figs 1, 2); abdomen of male more slender, ratio length of body / width of abdomen 2.15 (Figure 3) and deltg VII angularly produced posterolaterally (Figs 11, 12)	***heissi* (Bai et al., 2006), comb. n.**
–	Antennae shorter, approx. 1.9 times as long as width of head, anterolateral lobes of pronotum wider and less produced (Figs 39, 41), abdomen of female evenly rounded (Figs 40, 41), abdomen of male wider, ratio length of body / width of abdomen 2.0 and deltg VII less produced and rounded (Figs 47, 48)	***hainanensis* sp. n.**
3	Pronotum narrower 2.86 times wider than long, anterolateral lobes widely rounded (Figs 18, 20, 22, 24), median thoracic plate of meso- metanotum wider and lateral borders subparallel basally and at conical anterior part (Figs 18, 20, 22, 24)	***roundangulus* sp. n.**
–	Pronotum wider, more than three times as wide as long, anterolateral lobes narrower (Figs 60, 62, 64, 66), median thoracic plate of meso- metanotum narrower and distinctly leaf- shaped, diverging posteriorly, apical part attenuated anteriorly (Figs 60, 62, 64, 66)	***limuensis* sp. n.**

#### Paralibiocoris heissi

Taxon classificationAnimaliaHemipteraAradidae

(Bai, Yang & Cai, 2006) comb. n.

Figs 1–4 , 5–17

Libiocoris heissi Bai, Yang & Cai 2006: 41, figs 1, 3–7 (CAU).
Libiocoris sinensis Bai, Yang & Cai 2006: 43, figs 2, 8–12 (CAU) syn. n.

##### Type material.

Holotype (♀): China, Hainan, Baisha, Yinggeling, 1050 m, 10.IX.2005, L. S. Chen leg. (EMIH). **Additional material examined.** ♂, China, Hainan, Baisha,Yinggeling, 950 m, 2.VIII.2007, Bai X.S.; ♂, China, Hainan, Wuzhi mountain, 8.V.2008, Bai X.S.; ♀, China, Hainan, Baisha,Yinggeling, 950m, 2.VIII.2007, Bai X.S.; 2♀, China, Hainan, Ledong, Jianfengling, 900 m, 21.VII.2004, Wu Jie (EMIH, CAU); ♂,♀ China, Baisha, / Yinggeling 1200 m / 19°03'16"N, 109°33'53"E /2.VIII.2007, Bai X.S. (CEHI ex CAU).

As both taxa were described on single females and males are now available, the holotype of *heissi* is redescribed and additional features of the male added.

##### Diagnosis.

As generic description.

##### Redescription.

Apterous female, incrustation removed to recognise dorsal structures.

*Head.* Slightly longer than wide across eyes (1.0/0.9); clypeus short reaching basal one-third of first antennal segment, strongly raised anteriorly, with tubercle near apex; genae slightly produced over clypeus; antenniferous tubercles short, dilated, apices acute, diverging anteriorly; antennae 2.1 times as long as width of head across eyes, length of antennal segments I to IV = 0.65, 0.30, 0.65, 0.30; eyes small, not protruding; postocular tubercles small but distinct, not reaching outer margin of eyes; postocular borders behind tubercles straight and converging to constricted collar; vertex with Y-shaped granulate carina flanked by two(1+1) large, ovate infraocular callosities; rostrum short, rostral groove wide and deep, closed posteriorly.

*Pronotum*. 2.8 times as wide as long (1.4/0.5); collar narrow, anterolateral angles produced forward beyond collar as two (1+1) large, blunt, granulate lobes; disc with a longitudinal median furrow flanked by ovate callosities; posterior margin of pronotum slightly convex posteriorly, separated from mesonotum by a deep furrow.

*Mesonotum.* Wider than pronotum, separated from metanotum by two (1+1) deep furrows laterally; across meso- and metanota medially with an elongate, subpentagonal bottle-shaped ridge, 1.53 times as long as wide (0.87/0.57), subrounded anteriorly and truncate posteriorly, smooth and without longitudinal sulcus; lateral of median ridge disc with four (2+2) longitudinal sclerites, lateral margins granulate.

*Metanotum.* Wider than mesonotum; separated from fused mtg I+II by a slightly sinuate thin sulcus; lateral of median ridge with two (1+1) large subtriangular callosities, 2 (1+1) longitudinal ridges lateral of callosity discs, lateral margins granulate, similar to those of mesonotum.

*Abdomen*. Mtg I and II completely fused, depressed at middle, there with a median longitudinal ridge laterally separated by deep furrows from wide oblique lateral plates, sloping posteriorly and sideways, further laterally with two (1+1) large subtriangular depressions; tergal plate with a slightly raised median ridge on mtg III, a pentagonal elevation on mtg IV then tapering posteriorly.

*Venter.* Sterna III to VI raised along posterior border, depressed along anterior border, and with triangular, smooth spots medially, flanked by two (1+1) large, transversely ovate depressions, these bearing two (1+1) round callous spots; laterally four (2+2) smaller round callous spots present; spiracles II ventral, III–VIII lateral and visible from above.

*Legs.* Long and slender, without spines, preapical comb on fore tibia present, femora subcylindrical, claws with fine pulvilli.

***Male.*** Morphological features similar to female but of smaller size. Head as long as wide across eyes; median plate of meso- metanotum more elongate 1.82 times as long as wide (0.73/0.40); mtg VII strongly elevated posteriorly; reflexed vltg VII forming triangular lobes posterolaterally, ventral side with a distinct oblique smooth callus, reaching lateral margin; ptg VIII short and clavate much shorter than cordate pyriform pygophore (Figs 13, 14); parameres slender (Figs 15–17).

##### Measurements

[in mm, ♂(n = 2)/♀ (n = 3), holotype in parentheses]. Body length 4.4-4.45/5.1-5.8 (5.6); maximal width of abdomen 2.05-2.2/2.65-3.05 (3.05). Head length 0.8-0.85/0.9-1.05 (1.0), width 0.8/0.8-0.95 (0.9). Pronotum length 0.4/ 0.45-0.5 (0.5), width 1.15-1.2/1.3-1.45 (1.4). Mesonotum width 1.5-1.6/1.7-1.95 (1.8). Metanotum width 1.8-1.9/2.05-2.3 (2.25). Length of antennal segments I–IV = 0.60, 0.25, 0.60, 0.30/0.6-0.7, 0.3-0.35, 0.6-0.7, 0.3-0.35 (0.65, 0.30, 0.65, 0.30).

##### Distribution.

China (Hainan).

##### Comments.

As the generic characters and diagnosis are valid for all hereafter described new congeneric taxa, common features are not repeated except those differing in structure of thoracic median plate, size, and measurements distinctive for the specific taxa.

#### Paralibiocoris roundangulus sp. n.

Taxon classificationAnimaliaHemipteraAradidae

http://zoobank.org/7B6A48C4-B46F-49A5-BE47-635F083B0DFA

Figs 18–21 , 22–30 , 31–38

##### Type material.

Holotype: ♂, China, Hainan, Jianfeng, Tianchi, 810 m, 16.VIII.2007, Zhang & Bai (EMIH). **Paratypes.** 2♂, China, Hainan, Jianfeng, Tianchi, 810 m, 16.VIII.2007, Zhang & Bai; ♂, China, Hainan, Tongzha, Wuzhishan, 6.V.2009, Zhang & Yang; 2♀, China, Hainan, Jianfeng, Beiganxian, 820 m, 9.VIII.2007, Bai, X. S. (EMIH); 3♂, 3♀ collected with holotype (CEHI ex CAU).

##### Diagnosis.

General aspect similar to *Paralibiocorisheissi*, but distinguished from it by a wider pronotum, 2.86 times as wide as long (2.80 in *P.heissi*), anterolateral lobes widely rounded (narrow and more produced), shorter antennae 1.79 times as long as width of head (2.1). *Paralibiocorisroundangulus* sp. n. differs from *P.hainanensis* sp. n. and *P.limuensis* sp. n. by a different shape of the median ridge of meso- and metanotum (Figs 22, 24) and shorter antennae which are 1.79 times as long as width of head (1.89 and 1.82 respectively).

**Figures 18–21.**
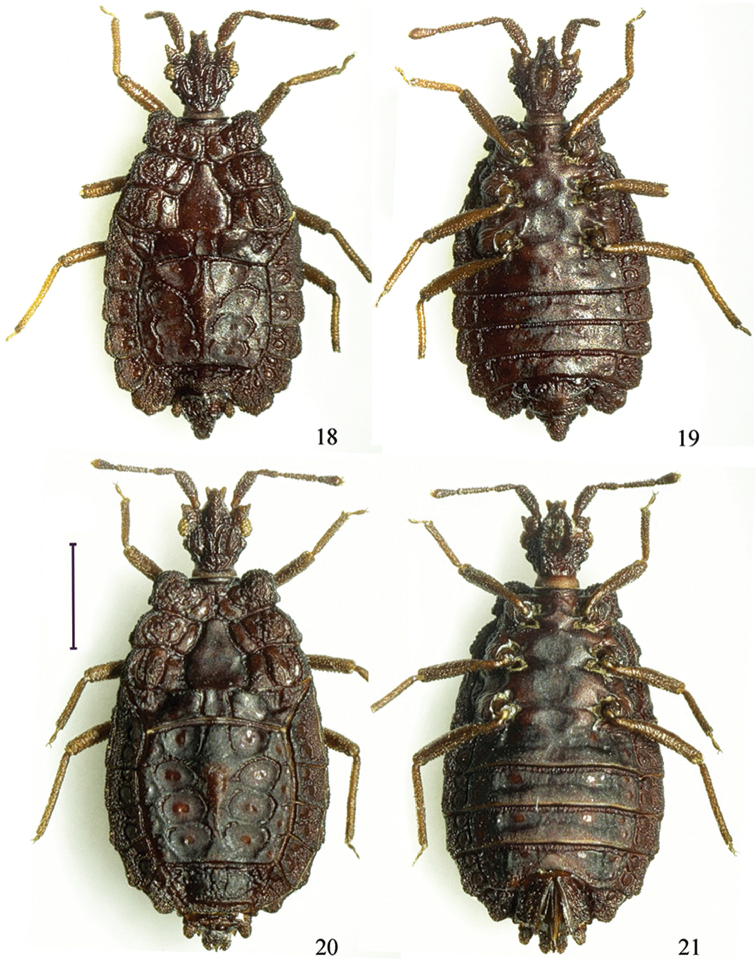
*Paralibiocorisroundangulus* sp. n. Holotype male (**18, 19**) dorsal and ventral view; female (**20, 21**) dorsal and ventral view. Scale bar: 1 mm.

##### Description.

**Male.** Basic morphological structures as of *P.heissi*. *Head.* As long as wide across eyes (0.82/0.82); antennae 1.79 times as long as width of head across eyes, length of antennal segments I to IV = 0.48, 0.24, 0.44, 0.31.

*Pronotum.* 2.86 times as wide as long (1.23/0.43); collar narrow; anterolateral angles produced forward beyond collar as two (1+1) widely rounded granulate lobes; disc with a longitudinal median furrow flanked by 2 (1+1) large, subtriangular and smaller callosities; lateral margin granulate.

*Mesonotum.* Wider than pronotum (1.60/1.23); separated from metanotum by two (1+1) deep furrows laterally; across meso- and metanota medially with an elongate, smooth bottle-shaped plate similar to *P.heissi*, 1.56 times as long as wide (0.67/0.43).

*Metanotum.* Wider than mesonotum (1.83/1.60); separated from mtg I by a slightly sinuate thin sulcus.

*Abdomen.* Mtg I and II completely fused, disc with a wide, smooth rectangular plate at middle flanked by two (1+1) large oblique plates, sloping posteriorly and sideways, laterally with two (1+1) small subtriangular depressions; deltg II and III fused, the following separated by fine sulci; posterolateral angles of deltg V to VII progressively angularly protruding; paratergites clavate, short, not reaching beyond posterolateral angles of deltg VII; pygophore elongate cordate, surface rugose (Figs 34, 35); parameres slender (Figs 36–38).

*Venter.* Sterna III to VI raised along posterior border, depressed along anterior border, and with triangular, smooth spots medially, flanked by 2 (1+1) shallow, transversely ovate depressions, these bearing 2 (1+1) round callous spots; 4 (2+2) smaller round callous spots present laterally; vltg VII with a small callus near spiracle VII; spiracles II ventral, III–VIII lateral and visible from above.

**Female.** Morphological features similar to male but of larger size; head slightly longer than wide across eyes (0.93/0.87); length of antennal segments I to IV = 0.48, 0.24, 0.44, 0.31; pronotum wider than long (1.47/0.43); width of mesonotum 1.90; width of metanotum 2.16, anterior lobe of median plate across meso- and metanota truncate, 1.2 times as long as wide (0.78/0.65); mtg VII moderately elevated posteriorly, the posterolateral angles forming triangular lobes; ptg VIII lobiform, reaching basal half of segment IX.

**Figures 22–30.**
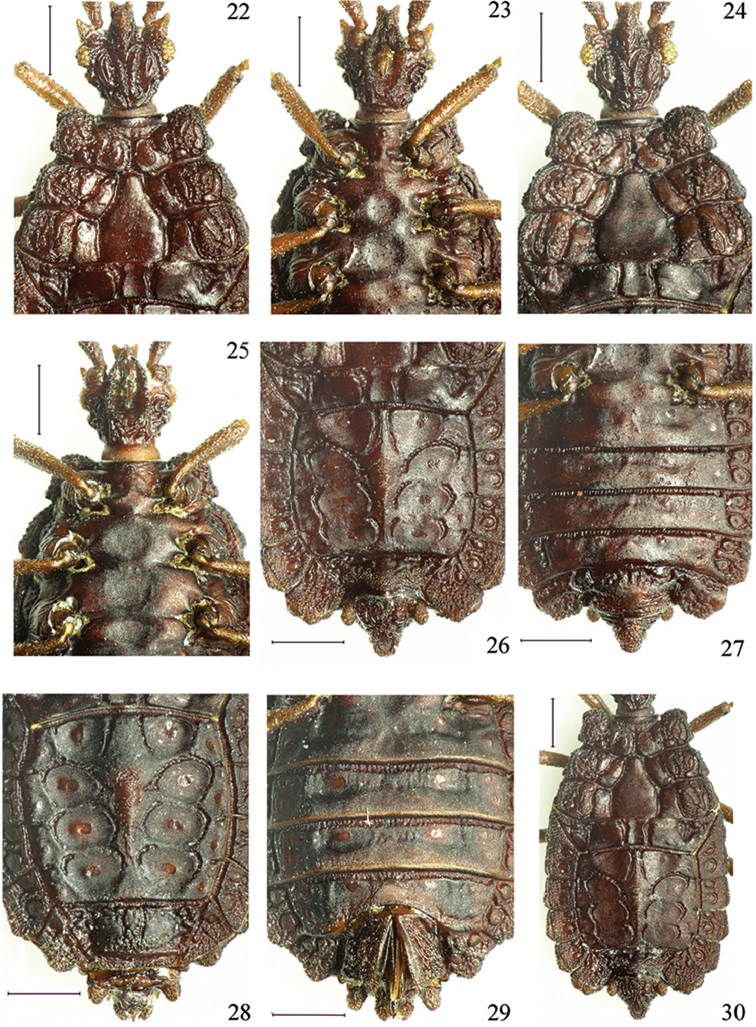
*Paralibiocorisroundangulus* sp. n. Holotype male (**22, 23, 26, 27, 30**) dorsal and ventral thorax and abdomen; female (**24, 25, 28, 29**) dorsal and ventral thorax and abdomen. Scale bars: 0.5 mm (**22–30**).

**Figures 31–38.**
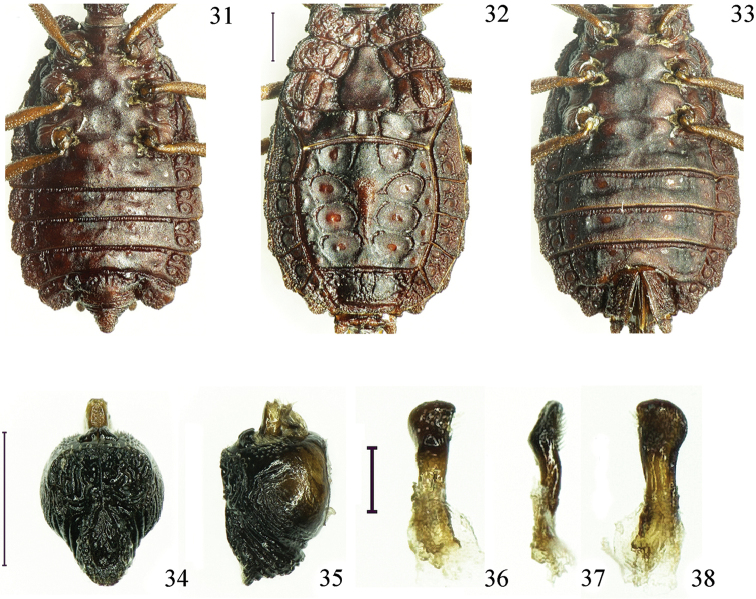
*Paralibiocorisroundangulus* sp. n. Holotype male (**31**) ventral thorax and abdomen; female (**32, 33**) dorsal and ventral thorax and abdomen; pygophore dorsal and lateral view (**34, 35**); right paramere in three positions (**36, 37, 38**). Scale bars: 0.5 mm (**31–35**), 0.1 mm(**36–38**).

##### Measurements.

[in mm, ♂(n = 4)/♀ (n = 2), holotype in parentheses]. Body length 4.1-4.15/4.9-5.1 (4.1); maximal width of abdomen 2.05-2.1/2.55-2.75 (2.1). Head length 0.82/0.9-0.93 (0.82), width 0.82/0.8-0.87 (0.82). Pronotum length 0.43/0.43 (0.43), width 1.23/1.4-1.47 (1.23). Mesonotum width 1.55-1.6/1.85-1.90 (1.60). Metanotum width 1.83-1.9/2.1-2.16 (1.83). Length of antennal segments I–IV = 0.48, 0.24, 0.44, 0.31/0.48, 0.24, 0.44, 0.31 (0.48, 0.24, 0.44, 0.31).

##### Etymology.

The name of species refers to the widely rounded anterolateral angles of pronotum.

##### Distribution.

China (Hainan).

#### Paralibiocoris hainanensis sp. n.

Taxon classificationAnimaliaHemipteraAradidae

http://zoobank.org/0E59DCF2-1EE8-4D07-90FF-9F14731E277F

Figs 39–42 , 43–51 , 52–59

##### Type material.

Holotype (♂): China, Hainan, Jianfeng, Tianchi, 810 m, 16.VIII.2007, Zhang & Bai; (EMIH). Paratypes: 2♂, China, Hainan, Changjiang, Bawangling, 13.IX.2008, Zhang W. J.; 2♂, 3♀ China, Hainan, Jianfeng, Tianchi, 810 m, 16.VIII.2007, Zhang & Bai; 3♂, China, Hainan, Tongzha, Wuzhishan, 6.V.2009, Zhang & Yang; ♂, China, Hainan, Wanning, Shimeiwan, 12.VIII.2007, Bai, X. S.; ♀, China, Hainan, Jianfeng, Nanya, 644 m, 22.VIII.2007, Bai, X. S. (EMIH); 2♂,2♀ collected with holotype (CEHI ex CAU).

##### Diagnosis.

General aspect similar to *Paralibiocorisheissi*, but distinguished from the latter by the wider pronotum, 2.91 times as wide as long (2.80 in *P.heissi*) and more rounded less produced anterolateral lobes (produced and blunt), shorter antennae 1.89 times as long as width of head (2.1) and by posterolateral angles of deltg V to VII slightly protruding and rounded in female. *Paralibiocorishainanensis* sp. n. differs from *P.roundangulus* sp. n. and *P.limuensis* sp. n. by a different shape of the median ridge of meso- and metanotum (Figs 43, 45 vs. Figs 22, 24 and Figs 64, 66, respectively) and antennal segment I as long as III (III shorter than I).

**Figures 39–42.**
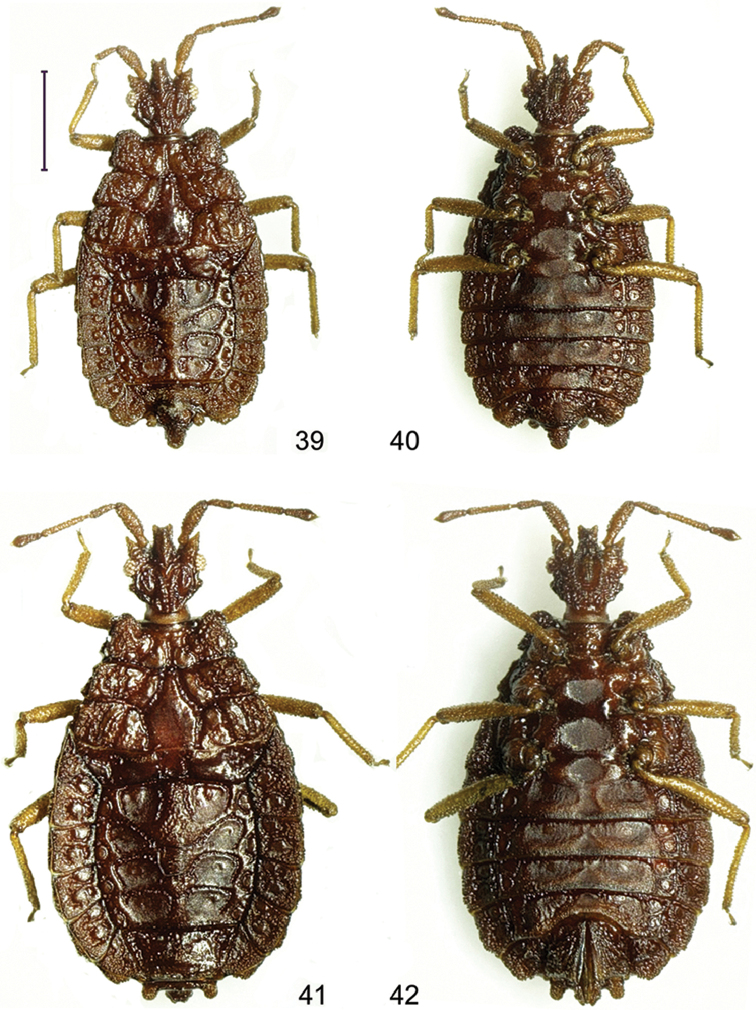
*Paralibiocorishainanensis* sp. n. Holotype male (**39, 40**) dorsal and ventral view; female (**41, 42**) dorsal and ventral view. Scale bar: 1 mm.

##### Description.

**Male.** Basic morphological structures as of *P.heissi* and other congeners. *Head.* Slightly longer than wide across eyes (0.8/0.78); antennae 1.89 times as long as width of head across eyes, length of antennal segments I to IV = 0.47, 0.24, 0.47, 0.30.

*Pronotum.* 2.91 times as wide as long (1.25/0.43); collar narrow; anterolateral lobes produced forward beyond collar as two (1+1) widely rounded granulate lobes; disc with a longitudinal median furrow flanked by 2 (1+1) large, subtriangular and smaller callosities, lateral margin granulate, converging anteriorly.

*Mesonotum.* Wider than pronotum (1.60/1.25); separated from metanotum by two (1+1) deep furrows laterally; across meso- and metanota medially with an elongate, smooth bottle - shaped plate as *P.heissi*, 1.63 times as long as wide (0.70/0.43).

*Metanotum.* Wider than mesonotum (1.80/1.60); separated from mtg I by a slightly sinuate thin sulcus.

*Abdomen.* Mtg I and II completely fused, disc depressed at middle with a flat rectangular sclerite separated from lateral ovate plates by deep furrows; tergal plate with a slightly elevated granulate ridge which is widest on mtg III, sloping posteriorly; pygophore elongate cordate, surface rugose (Figs 55, 56); parameres slender (Figs 57–59).

*Venter.* Vltg VII with a small shiny callus, near spiracle VII; spiracles II ventral, III–VIII lateral and visible from above.

**Female.** Morphological features similar to male but of larger size. Head slightly longer than wide across eyes (0.80/0.78); length of antennal segments I to IV = 0.46, 0.27, 0.46, 0.33; pronotum wider than long (1.27/0.43); width of mesonotum 1.60; bottle-shaped median thoracic plate 1.56 times as wide as long (0.67/0.43); width of metanotum 1.93.

**Figures 43–51.**
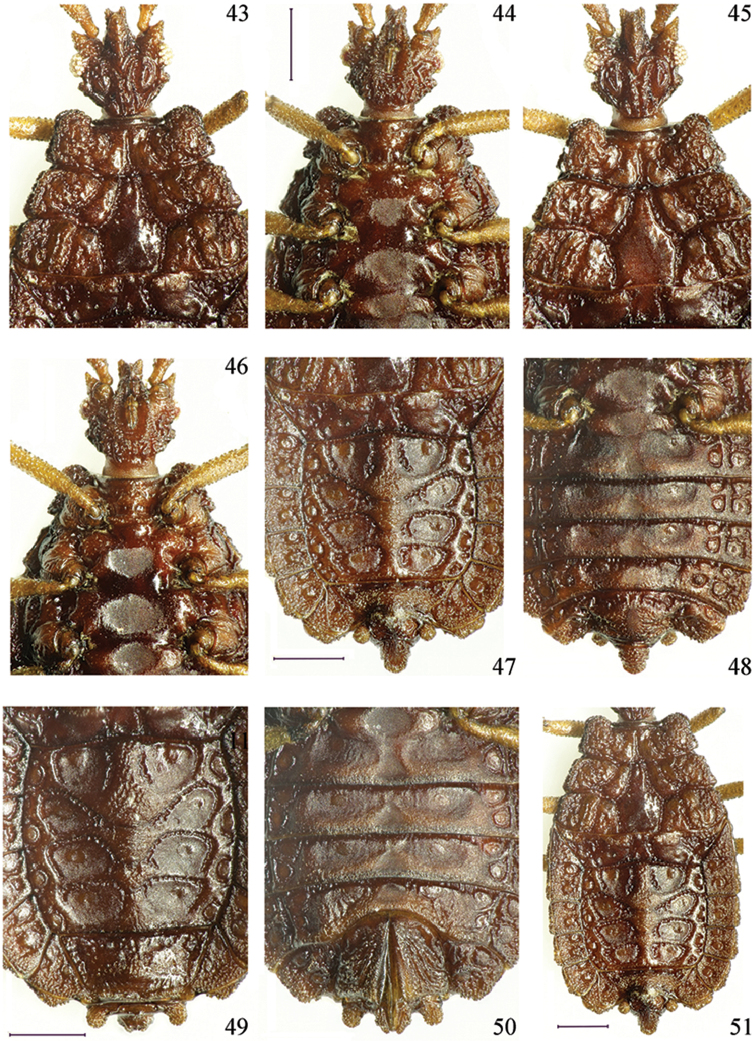
*Paralibiocorishainanensis* sp. n. Holotype male (**43, 44, 47, 48, 51**) dorsal and ventral thorax and abdomen; female (**45, 46, 49, 50**) dorsal and ventral thorax and abdomen. Scale bars: 0.5 mm.

**Figures 52–59.**
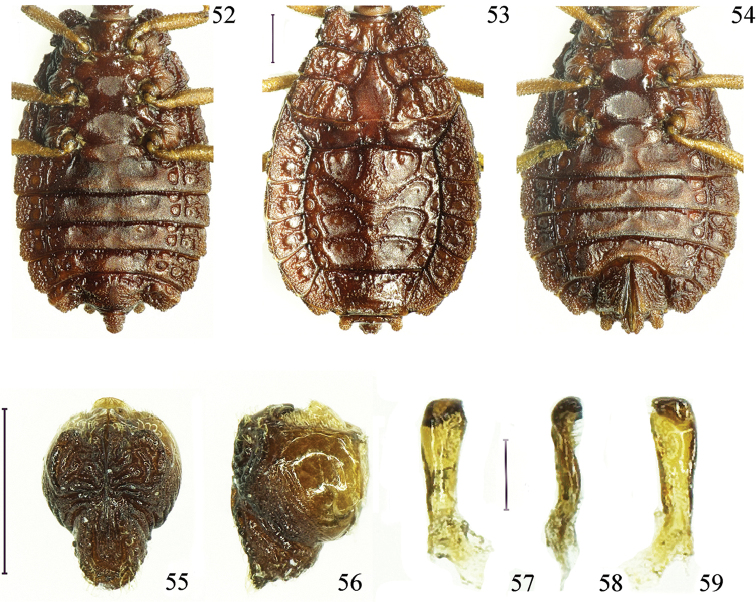
*Paralibiocorishainanensis* sp. n. Holotype male (**52**) ventral thorax and abdomen; female (**53, 54**) dorsal and ventral thorax and abdomen; pygophore dorsal and lateral view (**55, 56**); right paramere in three positions (**57, 58, 59**). Scale bars: 0.5 mm (**52–56**), 0.1 mm (**57–59**).

##### Measurements

[in mm, ♂ (n = 9)/♀ (n = 4), holotype in parentheses]. Body length 3.65-4.2/4.2-4.55 (4.2); maximal width of abdomen 1.75-2.1/2.2-2.4 (2.1). Head length 0.7-0.8/0.75-0.8 (0.8), width 0.65-0.78/0.7-0.78 (0.78). Pronotum length 0.35-0.43/ 0.4-0.45 (0.43), width 1.05-1.25/1.2-1.3 (1.25). Mesonotum width 1.4-1.6/1.45-1.6 (1.6). Metanotum width 1.5-1.8/1.65-1.9 (1.8). Length of antennal segments I–IV = 0.44-0.47, 0.24, 0.44-0.47, 0.3/0.47, 0.24-0.27, 0.47, 0.3-0.34 (0.47, 0.24, 0.47, 0.3).

##### Etymology.

The name refers to the Island of Hainan, the type locality.

##### Distribution.

China (Hainan).

#### Paralibiocoris limuensis sp. n.

Taxon classificationAnimaliaHemipteraAradidae

http://zoobank.org/10341EE3-0E54-4128-880D-304F8D2CD496

Figs 60–63 , 64–72 , 73–80

##### Type material.

Holotype (♂): China, Hainan, Limu, Montain, 6.V.2008, Bai, X. S.; (EMIH). **Paratypes.** 2 ♂, China, Hainan, Limu, Montain, 6.V.2008, Bai, X. S.; 2♀, China, Hainan, Limu, Montain, 6.V.2008, Bai, X. S. (EMIH).

##### Diagnosis.

**Figures 60–63.**
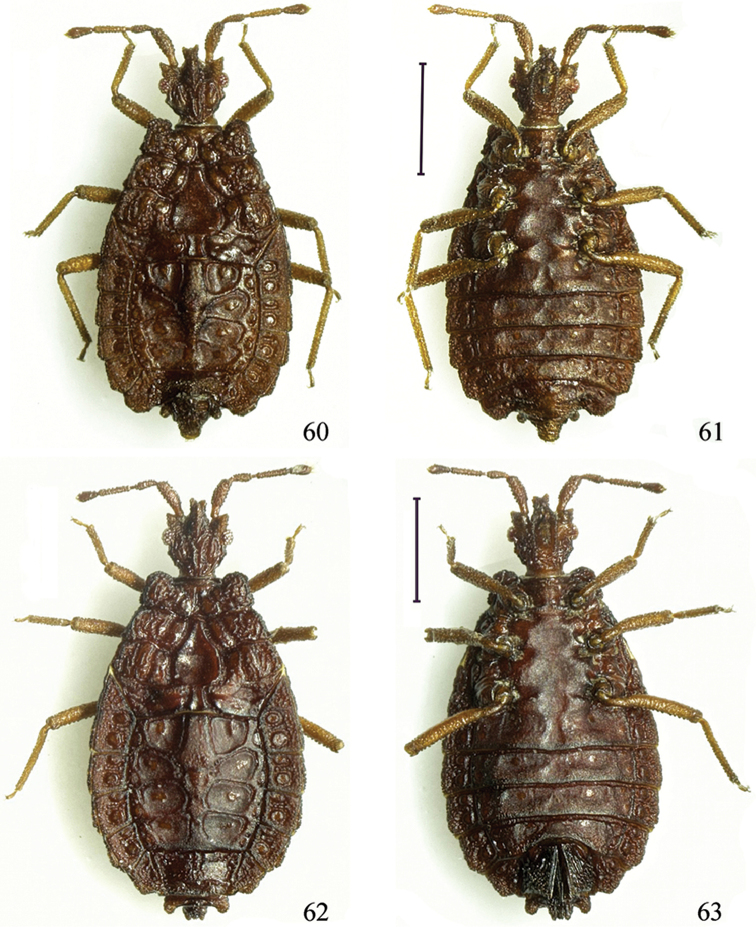
*Paralibiocorislimuensis* sp. n. Holotype male (**60, 61**) dorsal and ventral view; female (**62, 63**) dorsal and ventral view. Scale bars: 1 mm.

General aspect similar to *Paralibiocorisheissi*, but distinguished from the latter by wider pronotum 3.06 times as wide as long (2.80) and more rounded less produced anterolateral lobes (produced and blunt), shorter antennae 1.82 times as long as width of head (2.1) and by antennal segment I longer than III (of same lengths in *heissi*). *Paralibiocorislimuensis* sp. n. differs from *P.roundangulus* sp. n. and *P.hainanensis* sp. n. by a wider pronotum (3.06 vs. 2.86 and 2.91 respectively) and a leaf-like shape of the median ridge of meso- and metanotum (Figs 60, 62) and smaller size.

##### Description.

**Male.** Basic morphological structures as of *P.heissi* and other congeners. *Head.* Slightly longer than wide across eyes (0.75/0.68); antennae 1.82 times as long as width of head across eyes, length of antennal segments I to IV = 0.40, 0.20, 0.37, 0.27.

*Pronotum.* 3.06 times as wide as long (1.13/0.37); collar narrow; anterolateral lobes produced forward beyond collar as two (1+1) widely rounded granulate lobes, lateral margins converging anteriorly; structure of disc as in other congeners.

*Mesonotum.* Wider than pronotum (1.40/1.13); separated from metanotum by two (1+1) deep furrow laterally; across meso- and metanota medially with an elongate, anteriorly tapering leaf-like shaped plate, its surface slightly concave, 1.34 times as long as wide (0.63/0.47).

*Metanotum.* Wider than mesonotum (1.6/1.40); separated from mtg I by a slightly sinuate thin sulcus.

*Abdomen.* Mtg I and II completely fused, disc medially with a barrel-shaped sclerite resembling the leaf-stalk of the leaf - shaped ridge, separated from lateral ovate plates by deep furrows; tergal plate with a slightly elevated granulate ridge which is widest on mtg III, sloping posteriorly; pygophore elongate cordate, surface rugose (Figs 76, 77); parameres slender (Figs 78–80).

*Venter.* Vltg VII with a glabrous callus near spiracle VII; spiracles II ventral, spiracles III–VIII lateral and visible from above.

**Female.** Morphological features similar to male but of larger size; head slightly longer than wide across eyes (0.80/0.75); length of antennal segments I to IV = 0.44, 0.24, 0.37, 0.27; pronotum wider than long (1.17/0.40); width of mesonotum 1.6; width of metanotum 1.73; lateral margins of leaf-like median plate across meso- and metanota bisinuous, converging anteriorly to narrow apex, ratio length/width as of male (Figure 66); mtg VII moderately elevated posteriorly, surface rugose, posterolateral angles truncate.

**Figures 64–72.**
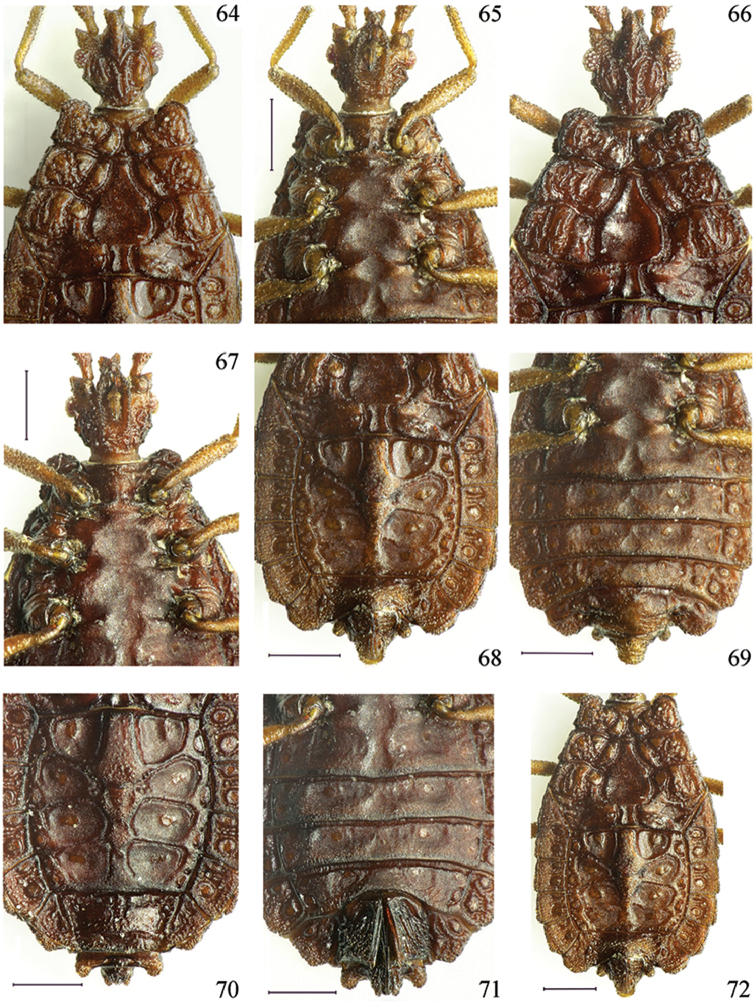
*Paralibiocorislimuensis* sp. n. Holotype male (**64, 65, 68, 69, 72**) dorsal and ventral thorax and abdomen; female (**66, 67, 70, 71**) dorsal and ventral thorax and abdomen. Scale bars: 0.5 mm.

**Figures 73–80.**
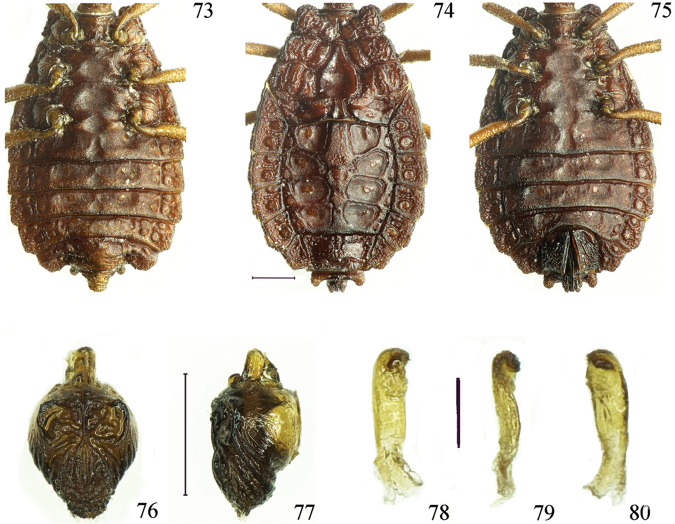
*Paralibiocorislimuensis* sp. n. Holotype male (**73**) ventral thorax and abdomen; female (**74, 75**) dorsal and ventral thorax and abdomen; pygophore dorsal and lateral view (**76, 77**); right paramere in three positions (**78, 79, 80**). Scale bars: 0.5 mm (**73–77**), 0.1 mm (**78–80**).

##### Measurements.

[in mm, ♂(n = 3)/♀ (n = 2), holotype in parentheses]. Body length 3.7-3.8/4.2-4.4 (3.8); maximal width of abdomen 1.76-1.9/2.2-2.3 (1.9). Head length 0.7-0.75/0.8 (0.75), width 0.65-0.68/0.7-0.75 (0.68). Pronotum length 0.35-0.37/ 0.4-0.45 (0.37), width 1.02-1.13/1.17-1.2 (1.13). Mesonotum width 1.3-1.4/1.5-1.6 (1.4). Metanotum width 1.5-1.6/1.73-1.8 (1.6). Length of antennal segments I–IV = 0.40, 0.20, 0.37, 0.27/0.4-0.44, 0.20-0.24, 0.37, 0.27 (0.40, 0.20, 0.37, 0.27).

##### Etymology.

The name of species reflects the locality of this new taxon.

##### Distribution.

China (Hainan).

**Map 1.**
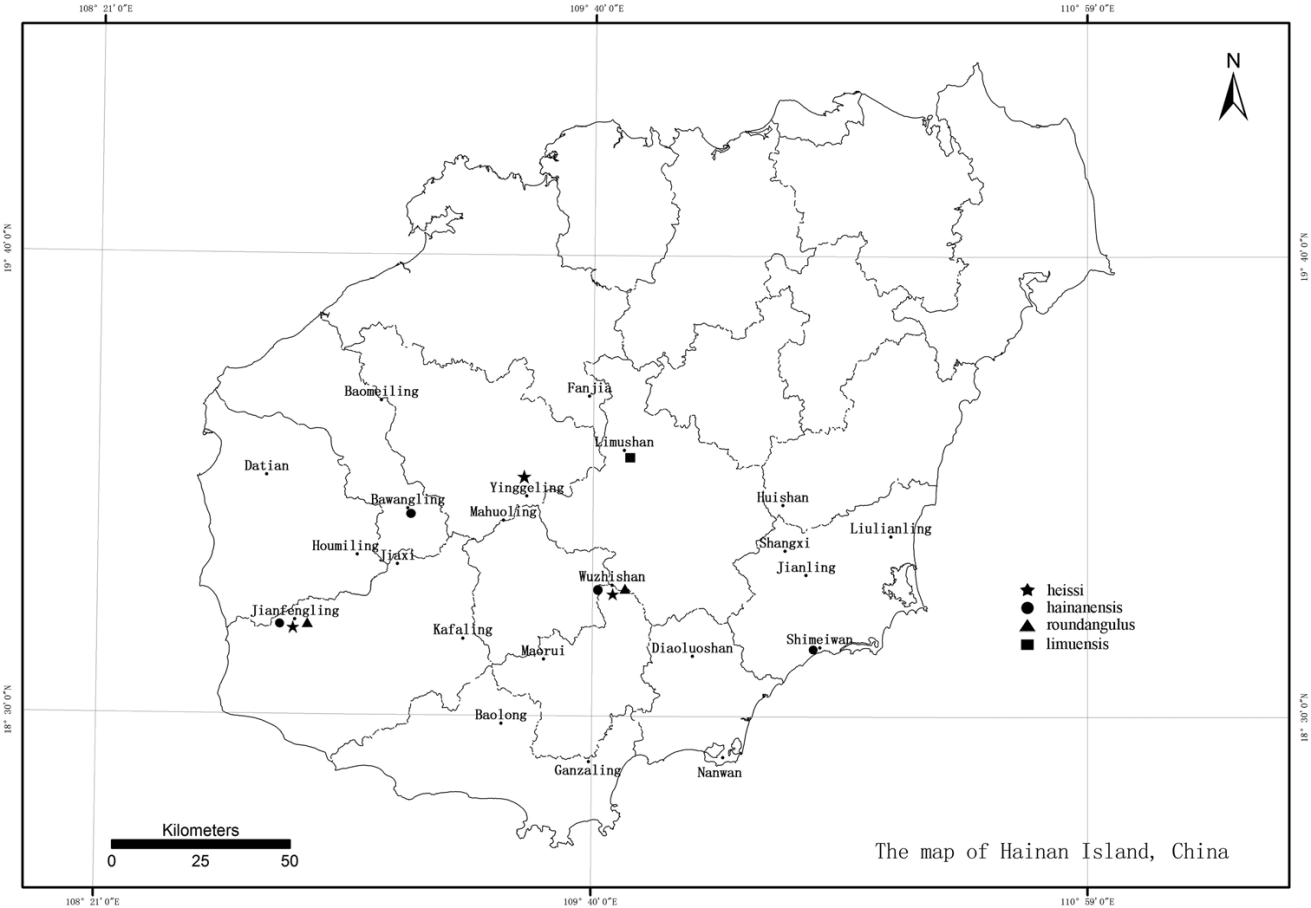
Distribution of *Paralibiocoris* in China, Hainan Island.

### The case of *Libiocorisindicus* Heiss, 1982

Figure 84

*Libiocorisindicus*Heiss 1982: 248 (description), figs 3–5 (HNHM, CEHI).

The species was described upon two males and a female from Meghalaya State in the north of India, and shares several morphological characters with *Libiocoris* (habitus, head and antennae, fusion of thoracic segments) and according to the (misleading) redescription of *Libiocoris* (based on their new species *L.antennatus* Usinger & Matsuda, 1959) also the position of spiracles II-VII placed laterally and visible from above. Due to this similarity it was “tentatively” assigned to *Libiocoris*.

**Figures 81–86.**
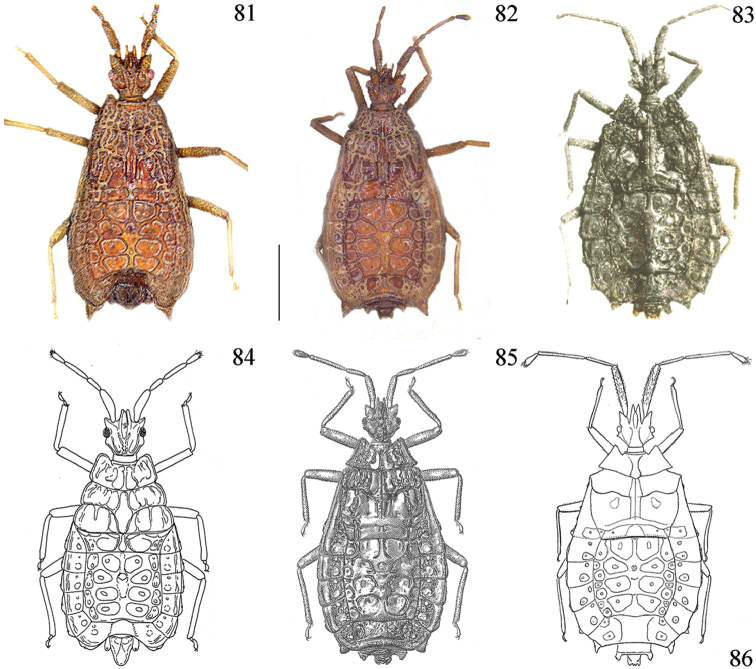
*Libiocoris*, habitus dorsal view. *L.poecilus* holotype male (**81**); ditto paratype female (**82**); *L.angulatus* holotype female (**83**); *L.indicus* holotype male (**84**)(after Heiss, 1982); *L.antennatus* holotype female (85) (after Usinger & Matsuda, 1959); *L.angulatus* holotype female, illustration in Usinger & Matsuda, 1959 with incorrect scale for antennae (**86**). Scale bar: 1 mm.

Re-examination of the paratype male of *indicus* has shown that it belongs neither to *Libiocoris* sensu Kormilev, 1957 (abbreviated L below) nor to *Paralibiocoris* gen. n. (abbreviated P below) showing following set of characters:

• position of spiracles: II-VII lateral and visible from above (not L, not P);

• fused deltg I+II shorter, reaching laterally only to metanotum (as P, not L);

• distinct glabrous oblique callus on vltg VII of male is lacking (as L, not P);

• fused median thoracic ridge of different shape (not L, not P);

• median thoracic ridge is elevated to sulcus of border metanotum, mtg I+II then sloping posteriorly (as P, not L);

• median ridge of abdomen raised along midline (as P, not L);

• dorsally reflexed vltg VII is subrectangular and not produced (as P, not L);

• shape of pygophore pyriform and produced posteriorly (as P, not L).

The position of spiracles, different from both *Libiocoris* and *Paralibiocoris*, is recognized as a diagnostic character used in Aradidae taxonomy for distinguishing genera [e.g., *Acaricoris* from *Kolpodaptera*; *Parapictinus* from *Mezira* (Usinger & Matsuda, 1959)] which supports a separate generic category for *indicus*. As other similar specimens from Vietnam and Japan present in our collections need to be included in a separate study, we refrain here from describing a new genus, but this species should be removed from *Libiocoris* sensu Kormilev, 1957. Inconsistencies and remarkable differences in the descriptions and illustrations of *angulatus*, *antennatus*, *lobatus*, and *pilosus* compared with *poecilus* raise questions about their generic assignment.

*Libiocoris* Kormilev, 1957

*Libiocoris* Kormilev, 1957 (1956): 390 (original description).

Type species *poecilus* by original designation, 391: figs 1–5 male HT, female AT, New Guinea (LP) HNHM (Figs 81, 82).

*Libiocorisantennatus* Usinger & Matsuda, 1958: 181, fig. 53 female HT, New Guinea (LA) MNHUK, PT in CEHI ex. coll. Kormilev (Figure 85).

*Libiocorisangulatus* Usinger & Matsuda, 1959: 84, fig. 84 female HT, New Guinea (LG) MCSM.

*Libiocorisangulatus* Heiss, 1989: 349, figs 12a, b (redescription, correction antennae) (Figs 83, 86).

*Libiocorislobatus*Kormilev 1968: 593 (description, no figure), New Guinea (LL); not seen.

*Libiocorispilicornis* Kormilev, 1972: 568, figs 9, 9A, New Guinea (LPC); not seen.

### Comparison of essential morphological characters and their differences

Anterior extension of fused deltg II+III reaching pronotum.

In LP present in types, not shown in Figure 1 of description; figured in LA, LG, LL?, figured only reaching mesonotum in LPC.

Position of spiracles denoted as + for visible from above and - for not visible. In LP II-III, VI-VII lateral +, IV ventral -, V sublateral -; LA II-VII lateral +; LG II-III lateral +, IV-V sublateral -; LL II-III, V-VIII lateral +, IV-V sublateral -; LPC II-VII lateral +.

Shape of median longitudinal ridge: in LP narrow with furrow; LA narrow with furrow; LG narrow, furrow figured; LL?; LPC posteriorly wider, without furrow.

As none of those female type specimens described after Kormilev’s definition of *Libiocoris* shares all these characters, it is questionable as to what might be a valid character state and what is due to variability, and whether they belong to the same generic category. This question can be resolved when further material will be available for study and barcoding.

## Supplementary Material

XML Treatment for Paralibiocoris

XML Treatment for Paralibiocoris heissi

XML Treatment for Paralibiocoris roundangulus

XML Treatment for Paralibiocoris hainanensis

XML Treatment for Paralibiocoris limuensis
